# Supplementary material for: Microbial iron oxide respiration coupled to sulfide oxidation
Source: Nature. 2025 Aug 27;646(8086):925–33. doi: 10.1038/s41586-025-09467-0 (PMC12545173; doi:10.1038/s41586-025-09467-0)
Supplement: Supplementary file 1 — Supplementary text including Supplementary Figs. 1–15 and references. [file 41586_2025_9467_MOESM1_ESM.pdf]

nature portfolio

https://doi.org/10.1038/s41586-025-09467-0

Supplementary information

Microbial iron oxide respiration coupled to sulfide oxidation

In the format provided by the authors and unedited

Supplementary Information

Microbial iron oxide respiration coupled to sulfide oxidation

Song-Can Chen, Xiao-Min Li, Nicola Battisti, Guoqing Guan, Maria A. Montoya, Jay Osvatic, Petra Pjevac, Shaul Pollak, Andreas Richter, Arno Schintlmeister, Wolfgang Wanek, Marc Mussmann, and Alexander Loy

Table of Contents

Supplementary Text **3**
A phylogenetic framework to discern functional homologs of sulfur-cycling proteins 3
Genomic prediction of iron(III) oxide-dependent sulfur oxidizers 6
Incubation of *D. alkaliphilus* with ferrihydrite and periodic spikes of FeS 10
Viability of *D. alkaliphilus* in ferrihydrite-only and sulfide-only incubations 11
Growth capacity of *D. alkaliphilus* 12
Incubation of *D. alkaliphilus* with ferrihydrite and a small amount of dissolved sulfide 13
Upregulated transcription of the multi-heme cytochrome gene DA_402 in *D. alkaliphilus* under ferrihydrite-amended conditions 13
Microscopy of *D. alkaliphilus* incubated with ferrihydrite and sulfide 14
Active transcription of genes involved in Wood Ljungdahl pathway under different growth conditions 15
Gating strategy of flow cytometry 17
Cell-specific metabolic rates of MISO 18
Geochemical phenomena consistent with the activity of MISO 18
Relevance of MISO in the global sulfur budget 18
References **20**

Supplementary Text

A phylogenetic framework to discern functional homologs of sulfur-cycling proteins

To improve sequence-dependent predictions of microbial sulfur metabolism, we performed systematic, phylogenetic analyses of 116 proteins that catalyse the cycling of inorganic and organic sulfur compounds (Table S1). This covered genes known to participate in major dissimilatory sulfur-cycling pathways, including sulfate reduction, sulfur oxidation, oxygen-dependent sulfur disproportionation, and metabolism of selected organosulfur compounds abundant in nature (i.e., dimethlysulfoniopropionate/dimethylsulfide and sulfoquinovose). The phylogeny-based method explicitly incorporates evolutionary path of function among protein family members, and provides some of the most accurate function inferences on uncharacterized proteins [1]. For each protein family, a maximum likelihood phylogeny was derived from sequences of biochemically characterized sulfur-cycling proteins, homologs with divergent functions, and further homologs retrieved from KEGG prokaryotic genomes (n=7942) (Fig. S1). After overlaying experimentally validated functions, gene neighbourhood patterns, and biochemical characteristics (i.e., catalytic residues) of all homologous proteins, the resulting annotated tree was manually scrutinised to pinpoint the clade(s) that may correspond to last common ancestor of functional homologs. Our criteria required high bootstrap support (>70%) of the assigned clades, with all descendants harbouring coherent biochemical and genomic features with experimentally validated proteins. This procedure yielded 180 robust monophyletic groups from 116 protein phylogenies, with each containing at least one experimentally validated member. Twenty seven protein families showed a polyphyletic evolutionary history, such as sulfite oxidase SorA (Table S1).

To leverage this manually curated dataset for large-scale genome searches, we reconstructed Hidden Markov Models (HMM) for each monophyletic group in the protein phylogenies, and compared their performance in detecting functional orthologs of sulfur-cycling proteins with existing HMMs from various sources. Benchmarking all HMMs against our manually curated dataset revealed that our phylogeny-derived HMMs showed the highest accuracy (Fig. S2). The wide range of specificities and sensitivities of existing HMMs in ortholog detection emphasized that reviewing the annotations yielded by existing HMMs is a prerequisite to reach more accurate and reliable prediction of sulfur metabolism proteins. The developed phylogenetic framework enables effective comparison of uncharacterized homologs with experimentally validated sulfur-cycling proteins in an evolutionary context, which facilitates their functional curation and biological interpretation.

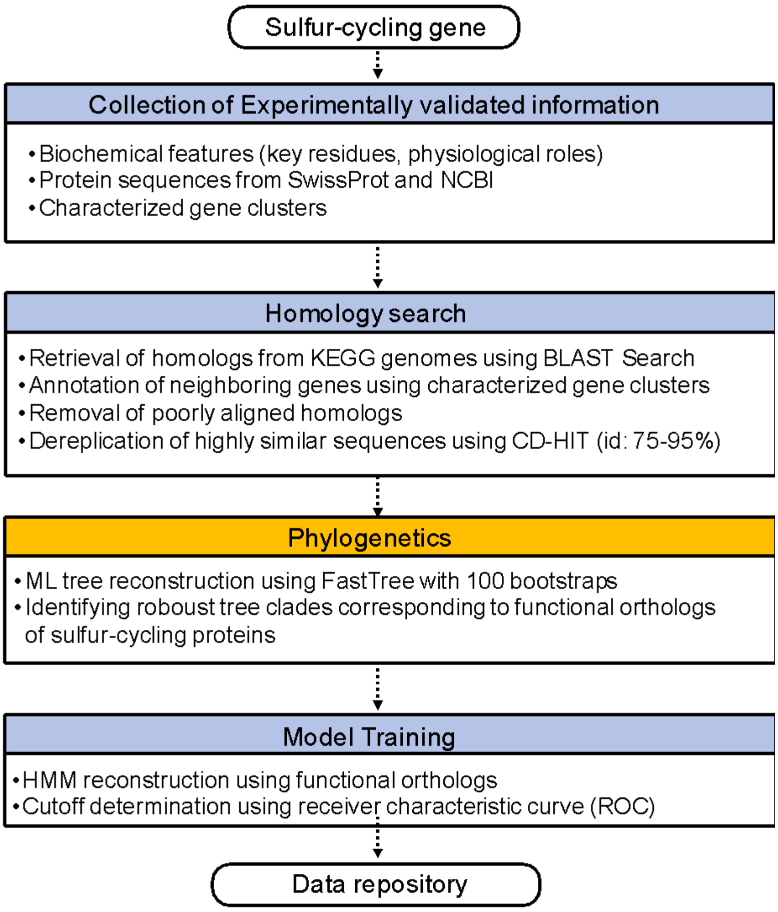

Figure S1 | Overview of the pipeline for reconstructing the phylogenetic framework of sulfur-cycling genes.

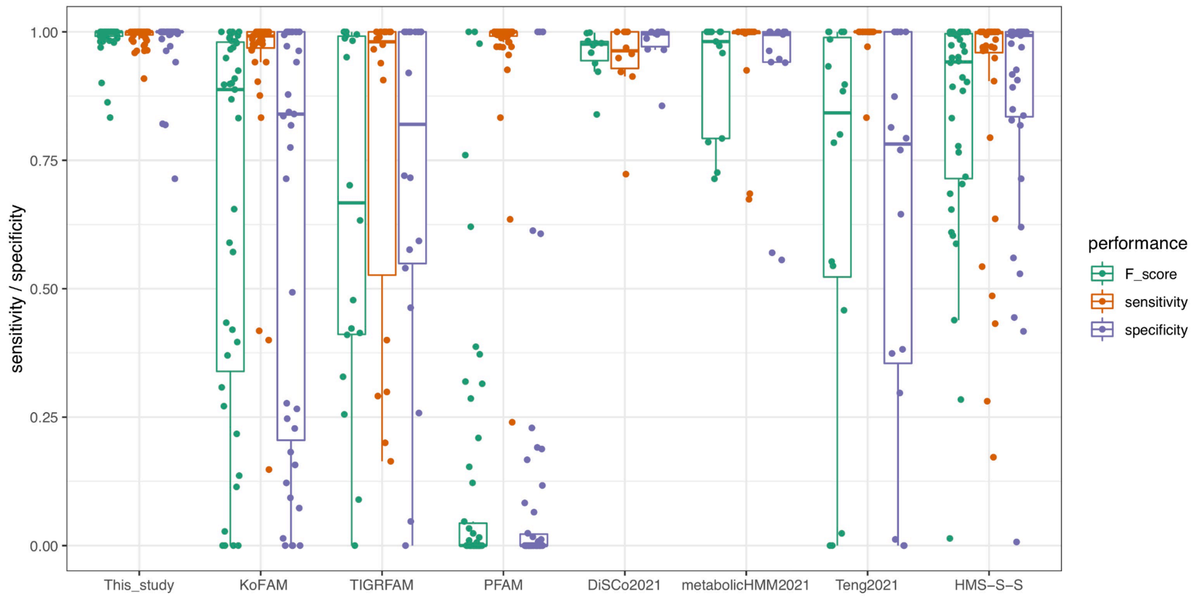

Figure S2 | Benchmarking of publicly available HMMs and HMMs developed in this study for sulfur-cycling genes against the dataset curated by phylogeny. HMMs were gathered from publicly available databases (KoFAM [2], TIGRFAM [3], and PFAM [4]) and from bioinformatics tools developed for detecting sulfur-cycling genes (DiSCo [5], metabolicHMM [6], Teng et al. [7], and HMS-S-S [8]). The performance of each HMM was assessed in terms of sensitivity, specificity, and F score.

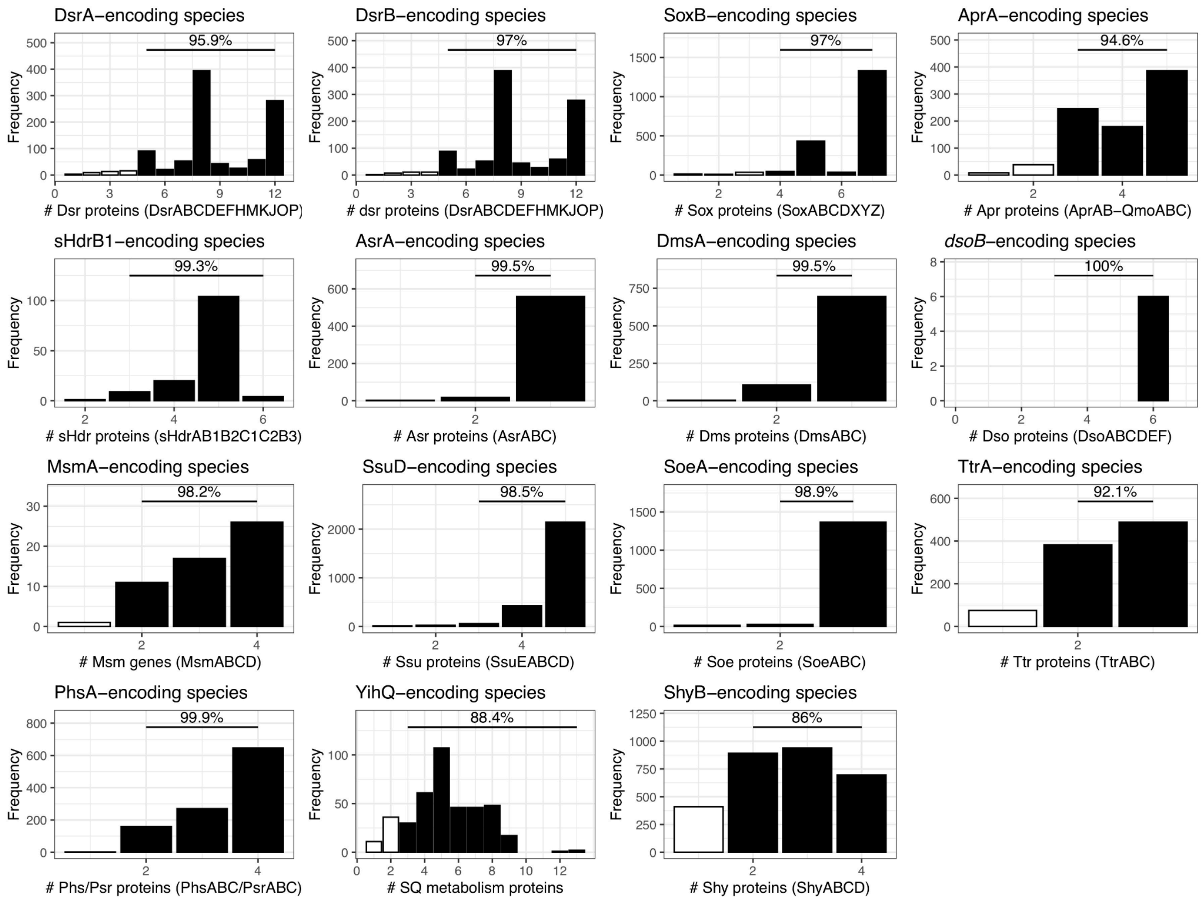

Figure S3 | Majority of genomes that encode a sulfur-cycling marker protein also encode additional enzymatic components for coordinated function. For each marker (e.g., DsrA), the number of additional sulfur metabolism proteins associated with it (e.g., DsrBCEFHMKJOP) was counted in each genome that contains the marker (e.g., DsrA-encoding genomes). The bar plot shows the frequency of the additional protein counts in the retrieved genomes. The horizontal line indicates the genomes with over 50% of proteins (e.g., five in the case of the Dsr system) for the full pathway or the complete multienzyme complex. The accumulated fraction (e.g., 95.9% for DsrA) of genomes harboring over 50% of proteins involved in the pathway or the enzymatic complex is shown above the line. Sulfur-cycling marker proteins that alone catalyze the specific sulfur redox transformation (e.g., sulfide:quinone oxidoreductase) were not analyzed.

Genomic prediction of iron(III) oxide-dependent sulfur oxidizers

The capability to couple sulfur oxidation with reduction of iron(III) oxides in GTDB species was predicted based on co-occurrence of genetic systems for dissimilatory sulfur oxidation (e.g., dissimilatory sulfite reductase (Dsr), sulfur-oxidizing heterodisulfide reductase-like complex sHdr, thiosulfate-oxidizing system Sox, sulfide:quinone reductase Sqr, and sulfide dehydrogenase Fcc), and iron(III) oxide reduction. The latter include porin-cytochrome complex [9–11], transmembrane complex MtrCAB [12], flavin-based extracellular electron transfer (EET) pathway (FmnAB-DmkAB-PplA-Ndh2-EetAB) identified in Gram-positive bacteria (e.g., *Listeria monocytogenes* [13]), outer membrane cytochromes (OMCs) involved in dissimilatory iron reduction and/or EET, such as OmcS/OmcF/OmcZ from *Geobacter* species [10,11,14], MmcA from methanogens [15], DFE_0450/DFE_0464 from *Desulfovibrio ferrophilus* IS5 [16], GACE_1845/GACE_1847 from *Geoglobus* species [17,18], and predicted OMCs from anaerobic methanotrophs [19,20] and putative electroactive microbes [21]. This screening procedure returned diverse members from 37 bacterial and archaeal phyla (Fig. S4-5). Prominent examples are from *Desulfobacterota* and *Gammaproteobacteria* (Fig. S4-5), including uncultivated members of *Desulfurivibrionaceae* and *Rhodoferax*. Phylogenetic analysis revealed the DsrAB sequences from *Desulfurivibrionaceae* form a monophyletic clade containing the characterized sulfide-oxidizer *Desulfurivibrio alkaliphilus* (Fig. S6). This suggests the uncultivated *Desulfurivibrionaceae* may operate DsrAB in reverse for oxidizing sulfide to sulfate, similar to their cultivated relatives *D. alkaliphilus* and cable bacteria [22,23]. The detection of dissimilatory iron reduction pathways in *Desulfurivibrionaceae*, such as those catalyzed by porin-cytochrome complex and OmcS, is supported by previous genome analysis of *D. alkaliphilus* [24,25]. Unlike *Desulfurivibrionaceae*, *Rhodoferax* spp. encode the genetic potential to oxidize sulfide to elemental sulfur using iron(III) oxides as electron acceptors (reaction 2 in the main text). The sulfide oxidation in these species could be facilitated by Sqr and Fcc. Phylogenetic analysis revealed their Sqr belongs to Sqr I (Fig. S7a), known to be involved in sulfur-based bioenergetics. Gene context analysis indicated *fcc* located in close proximity with *sox* genes (Fig. S7b), supporting their involvement in sulfur oxidation. The dissimilatory iron(III) reduction in *Rhodoferax* may be mediated by a transmembrane complex MtrCAB closely related to the complex from iron-reducing bacteria *Rhodoferax ferrireducens* (Fig. S7c) [26,27].

Beyond *Desulfobacterota* and *Proteobacteria*, members from other microbial lineages encode the potential to couple oxidation of sulfur compounds with the reduction of iron(III) oxides (Fig. S4-5), through the three reactions described in the main text. These include (1) members from *Chloroflexota*, *Bacteroidota*, and *Marinisomatota* encode the sHdr complex and predicted OMCs, which would facilitate reaction 1; (2) members from *Aquificota*, *Campylobacterota*, *Methylomicrabilis*, and *Nitrospirota* encode the Sox complex and *D. ferrophilus*-related and/or predicted OMCs, which would facilitate reaction 2; and (3) Sqr-encoding members from *Halobacteriota*, *Firmicutes*, *Bdellovibrionota*, *Bacteroidota*, and *Acidobacteriota* that also encode the flavin-based EET pathway, *Geoglobus*-related OMCs or predicted OMCs, and thus have the potential to catalyze reaction 3.

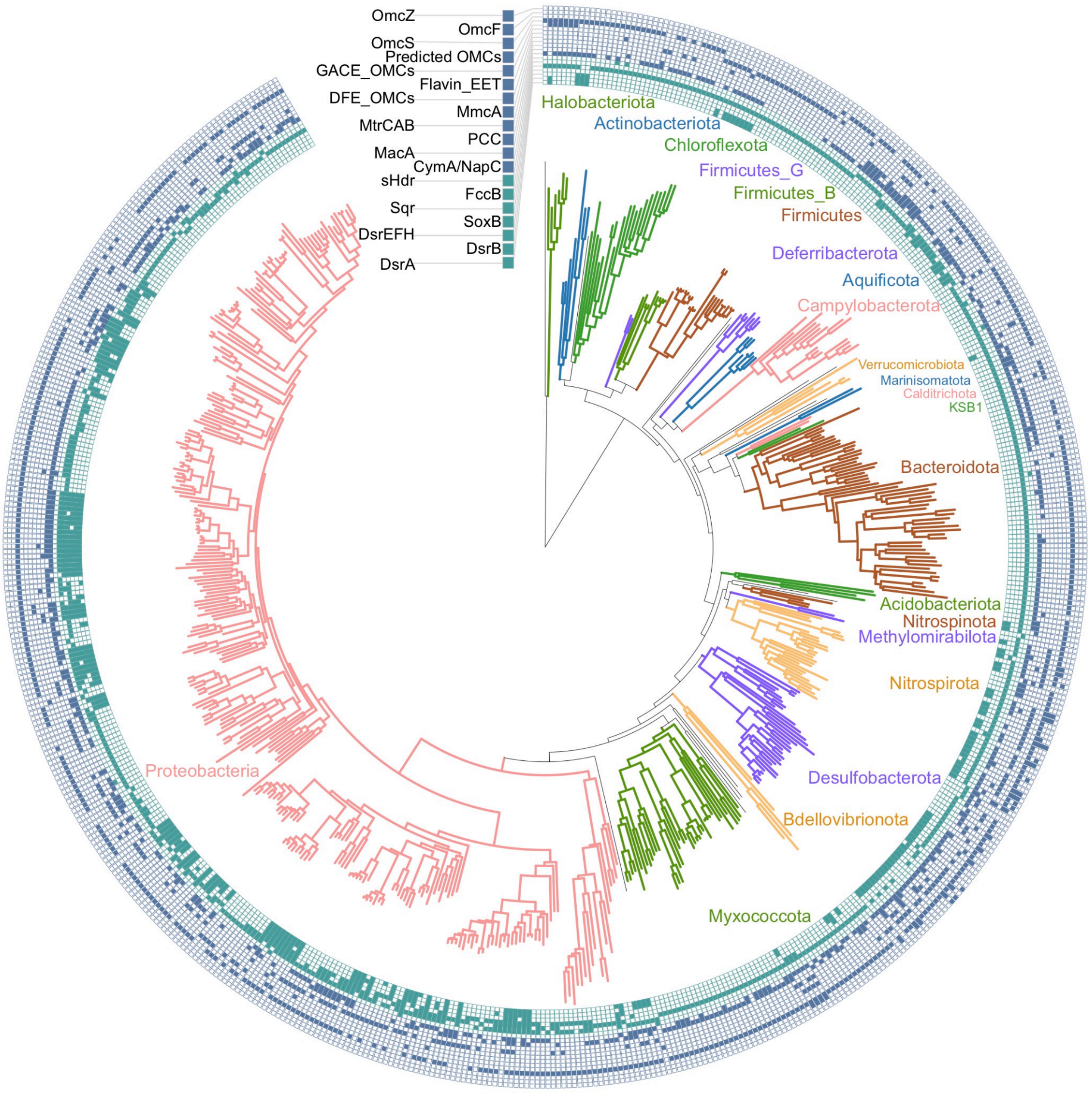

Figure S4 | **Phylogeny of microbes with the genomic potential for extracellular iron(III)-dependent oxidation of sulfur compounds.** The phylogenomic tree is pruned from the GTDB r95 bacterial and archaeal species tree. The presence/absence of metabolic pathways and/or key enzymes involved in dissimilatory sulfur oxidation (green) and dissimilatory iron(III) reduction (blue) is shown in the outer circles. DsrAB, dissimilatory sulfite reductase; DsrEFH, sulfur-relay system; SoxB, S-sulfosulfanyl-L-cysteine sulfohydrolase; Sqr, sulfide quinone reductase; FccB, sulfide dehydrogenase flavocytochrome subunit; sHdr, sulfur-oxidizing heterodisulfide reductase-like complex; CymA/napC, tetraheme quinol dehydrogenase; MacA, diheme cytochrome c involved in iron(III) reduction; PCC, porin cytochrome complex; MtrCAB, extracellular iron oxide respiratory system; MmcA, multiheme c-type cytochromes from *Methanosarcina acetivorans*; DFE_OMCs, outer-membrane multiheme c-type cytochromes (OMCs; DFE_0450 and DFE_0464) that enabled extracellular electron transfer (EET) between cells and insoluble minerals in *Desulfovibrio ferrophilus* IS5; Flavin_EET, the flavin-based EET pathway involved in iron reduction in Gram-positive bacteria (e.g., *Listeria monocytogenes*); GACE_OMCs, OMCs (GACE_1845 and 1847) involved in insoluble Fe(III) reduction in *Geoglobus* species; predicted OMCs, extracellular cytochromes that have >= 4 heme-binding sites; OmcS/F/Z, outer membrane cytochromes in *Geobacter* species.

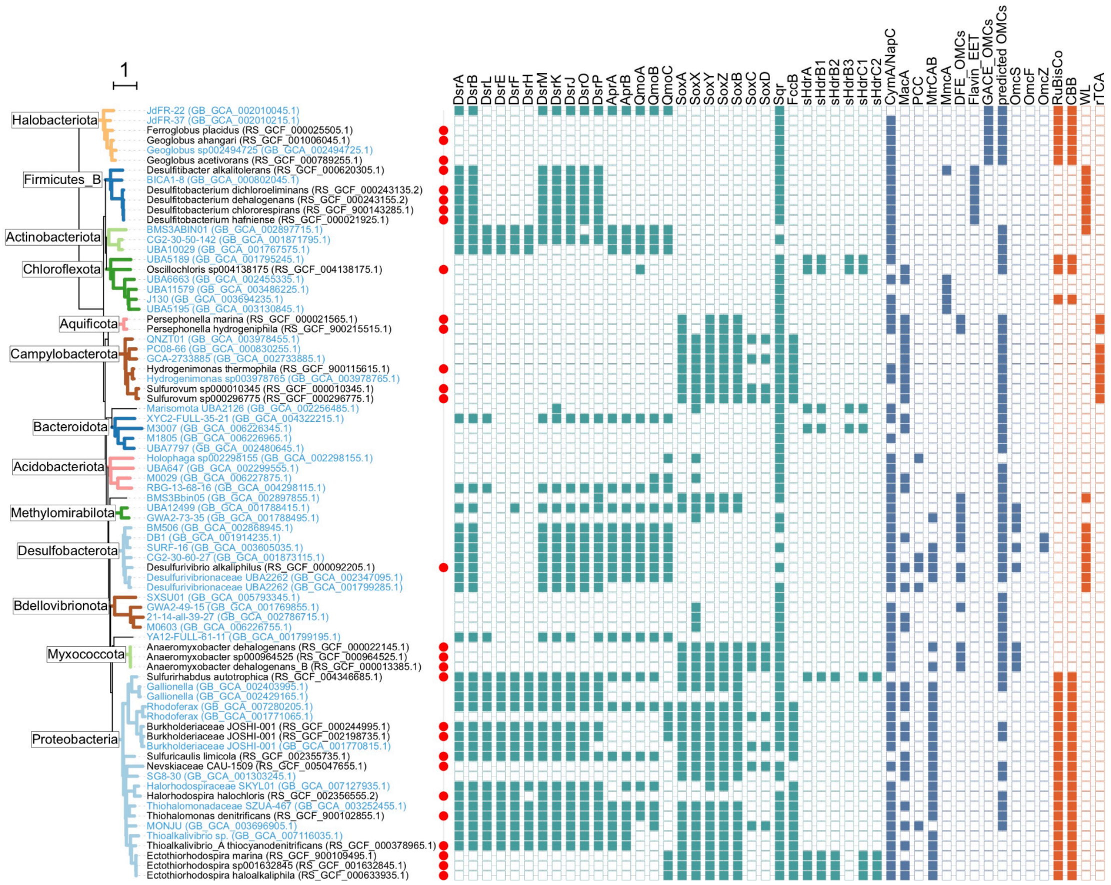

Figure S5 | Phylogeny of representative microbes with the metabolic potential for extracellular iron(III)-dependent oxidation of sulfur compounds. Representative taxa are selected from Figure S4 for visualization purposes. Pure cultures are highlighted with red circles and uncultured taxa depicted in blue. The presence/absence of metabolic pathways and/or key enzymes involved in dissimilatory sulfur oxidation (green), dissimilatory iron(III) reduction (blue), and carbon fixation (orange) is shown in the right panel. DsrAB, dissimilatory sulfite reductase; DsrL, NAD(P)H oxidoreductase; DsrEFH, sulfur-relay system; DsrMKJOP, [DsrC]-trisulfide reductase; AprBA, adenylylsulfate reductase; QmoABC, quinone-modifying oxidoreductase; SoxAX, L-cysteine S-thiosulfotransferase; SoxB, S-sulfosulfanyl-L-cysteine sulfohydrolase; SoxY, sulfur-oxidizing protein SoxY; SoxZ, sulfur-oxidizing protein SoxZ; SoxCD, sulfane dehydrogenase; Sqr, sulfide quinone reductase; FccB, sulfide dehydrogenase flavocytochrome subunit; sHdrAB1B2B3C1C2, sulfur-oxidizing heterodisulfide reductase-like complex; CymA/NapC, tetraheme quinol dehydrogenase; MacA, diheme cytochrome c involved in iron(III) reduction; PCC, porin cytochrome complex; MtrCAB, extracellular iron oxide respiratory system; MmcA, multiheme c-type cytochromes from *Methanosarcina acetivorans*; DFE_OMCs, the outer-membrane multiheme c-type cytochromes (OMCs; DFE_0450 and DFE_0464) that enabled extracellular electron transfer (EET) between cells and insoluble minerals in *Desulfovibrio ferrophilus* IS5; Flavin_EET, the flavin-based EET pathway involved in iron reduction in Gram-positive bacteria (e.g., *Listeria monocytogenes*); GACE_OMCs, OMCs (GACE_1845 and 1847) involved in insoluble Fe(III) reduction in *Geoglobus* species; predicted OMCs, extracellular cytochromes that have >= 4 heme-binding sites; OmcS/F/Z, outer membrane cytochromes in *Geobacter* species. RuBisCO, ribulose-bisphosphate carboxylase; CBB, Calvin-Benson-Bassham cycle; WL, Wood Ljungdahl pathway; rTCA, reverse tricarboxylic acid cycle.

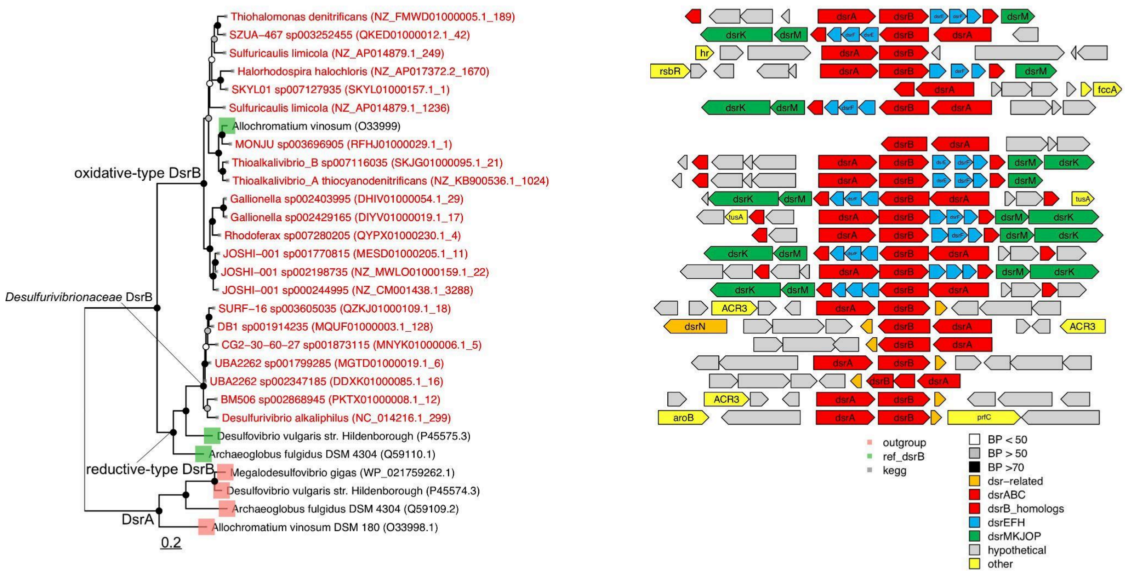

Figures S6 | Phylogeny and gene context of DsrB encoded in potential iron-dependent sulfur oxidizers. DsrB-encoded genomes are shown in red type. The DsrB homologs from the gammaproteobacterial lineages are placed within the clade spanned by oxidative-type DsrB from green-sulfur bacteria (e.g., *Prosthecochloris* and *Chlorobium*) and purple-sulfur bacteria (e.g., *Allochromatium vinosum*; green squares). The DsrB homologs from *Desulfurivibrionaceae* form a monophyletic clade within the reductive-type DsrB. DsrA phylogeny exhibits a similar branching pattern as DsrB, and is therefore not shown. Phylogeny of DsrB was rooted using DsrA sequences (shown with red squares) as outgroups. Biochemically validated sequences are highlighted with green squares. Branches with bootstrap value over 70%, between 50% and 70%, and below 50% are indicated by black, grey, and white circles, respectively. The scale bar indicates the number of amino acids substitution per site.

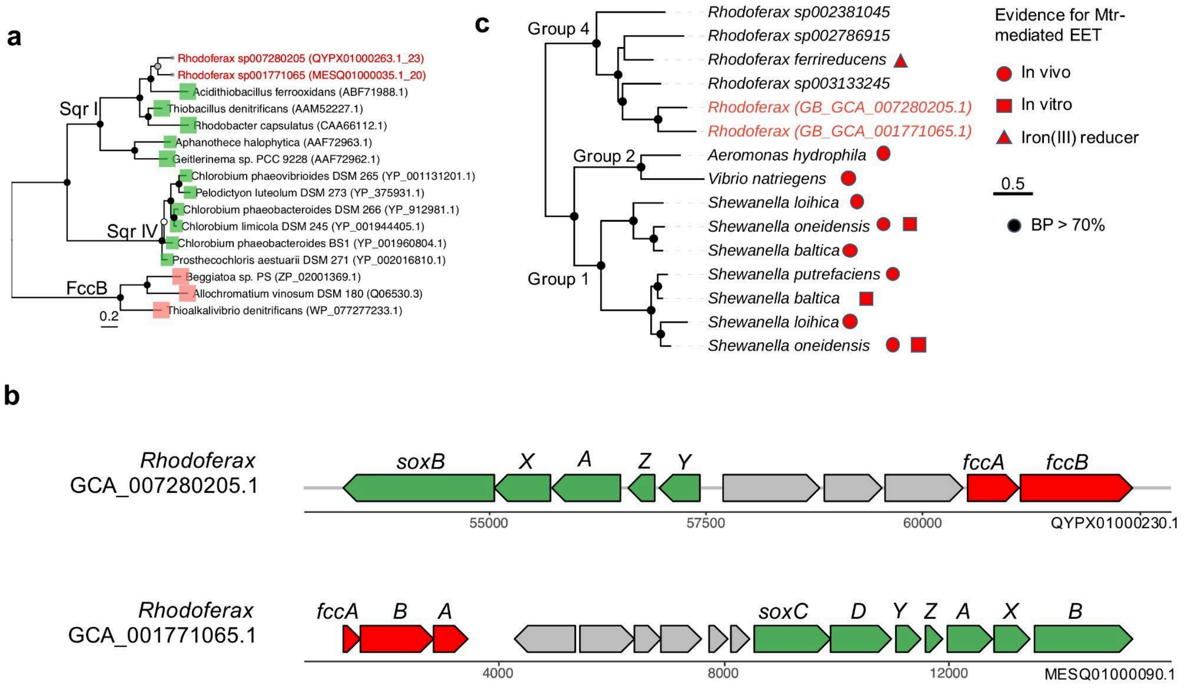

Figures S7 | Phylogeny and gene context of Sqr, Fcc, and MtrCAB encoded in uncultivated *Rhodoferax* species. a. The Sqr homologs from *Rhodoferax* spp. (highlighted in red) are affiliated to Sqr I [28,29]. Functionally verified Sqr and Sqr from well-characterized sulfide oxidizers are indicated by green squares. The phylogeny is rooted using protein sequences of FccB (sulfide dehydrogenase, flavocytochrome subunit) as the outgroup. Branches with bootstrap value over 70%, between 50% and 70%, and below 50% are indicated by black, grey, and white circles, respectively. The scale bar indicates the number of amino acids substitution per site. **b**. Gene context analysis shows *fccAB* (red arrows) from *Rhodoferax* clusters with *sox* genes (green arrows). **c**. Phylogeny of MtrCAB from *Rhodoferax*. The tree is reconstructed from concatenated alignment of three subunits of MtrCAB complex. The MtrCAB from uncultivated *Rhodoferax* species (highlighted in red) belong to Group 4 clade, as classified previously [27], and share close similarity with that encoded in the iron-reducing bacteria *Rhodoferax ferrireducens* [26]. Functionally characterized MtrCAB sequences from *Shewanella*, *Aeromonas*, and *Vibrio* belong to Group 1 and 2 clades, and branch sister to MtrCAB from *Rhodoferax*. Branches with bootstrap value over 50% are indicated by black circles. The scale bar indicates the number of amino acids substitution per site.

Incubation of *D. alkaliphilus* with ferrihydrite and periodic spikes of FeS

Periodic addition of FeS to *D. alkaliphilus* cultures with ferrihydrite led to continuous production of sulfate (Fig. S8a). In comparison, sulfate production was not observed in the biotic controls with only FeS or ferrihydrite, or in abiotic controls amended with FeS and ferrihydrite. Significantly more Fe(II) was produced by cultures amended with FeS and ferrihydrite incubated cells than in FeS-amended biotic and abiotic controls ($P < 0.01$; T-test) (Fig. S8b). This indicated active Fe(II) formation on top of Fe(II) added via the FeS spikes.

This incubation experiment was also used to benchmark the method for the measurement of HCl-extractable Fe(II) in the system containing both sulfides (e.g., dissolved sulfide or FeS) and ferrihydrite. Fe(II) measurement requires HCl treatment of the sample, during which sulfide liberated from FeS reacts with residual ferrihydrite. This step may generate additional Fe(II), leading to overestimation of Fe(II) as previously reported [30]. To estimate the effect of this reaction in our incubation experiment, we correlated the HCl-extractable Fe(II) in two treatments from our FeS/ferrihydrite incubation experiment. These include (1) the abiotic control fed with ferrihydrite and FeS and (2) the FeS-only control, both of which are free of biologically produced Fe(II). We reasoned that the FeS in abiotic control gets dissolved during the acidification step, with the released sulfide reacting with ferrihydrite that produces additional Fe(II) on top of FeS-Fe(II). Yet, the same reaction between ferrihydrite and acid-liberated sulfide does not occur in the FeS-only control. Correlation analysis of HCl-extractable Fe(II) in abiotic control vs. FeS-only control revealed a slope of 1.23 ± 0.07 (Fig. S9), indicating an overestimation of Fe(II) by 23 ± 7%. However, we argue this overestimation does not affect our conclusion because we interpreted most of Fe(II) data qualitatively. The only quantitative case was the stoichiometry during the FeS/ferrihydrite incubation. Correcting the observed stoichiometry (~1:6) by accounting for the overestimation of Fe(II) yielded ~1:4.9, which still supports the reverse electron flow for carbon fixation.

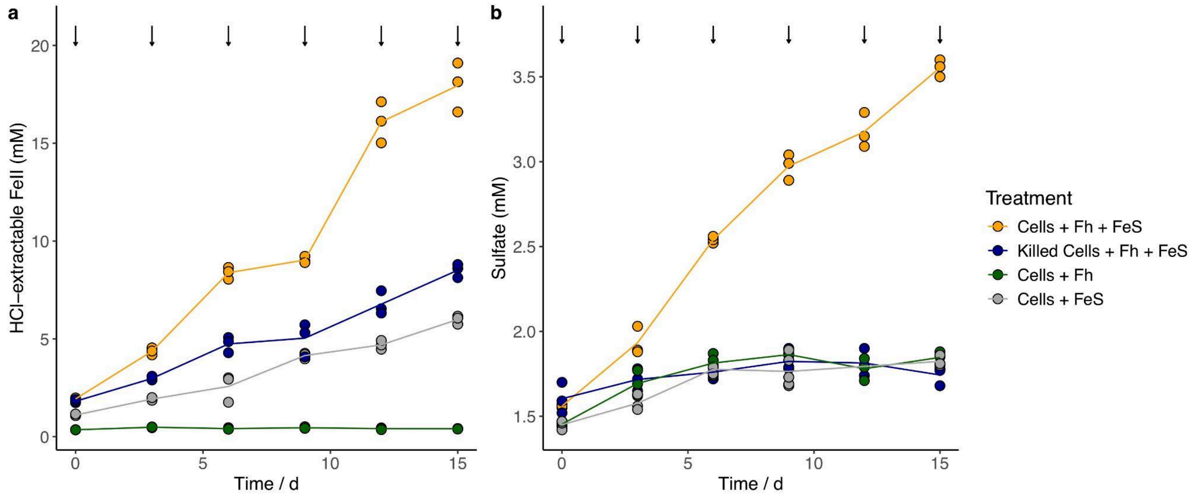

Figure S8 | Oxidation of FeS to sulfate coupled to reduction of ferrihydrite sustains survival of *D. alkaliphilus* cells. Triplicate cultures were used for each incubation condition. Sulfate production (**a**), HCl-extractable Fe(II) production (**b**) and cell number (**c**) during a 17-day incubation of *D. alkaliphilus* with ferrihydrite (Fh) and periodic spikes of FeS. Abiotic control incubations with FeS and Fh were performed with autoclaved cells. Biotic control incubations consisted of *D. alkaliphilus* cultures with only Fh or FeS. The arrows in **a** and **b** indicate additions of FeS (~ 1 mM) every three days.

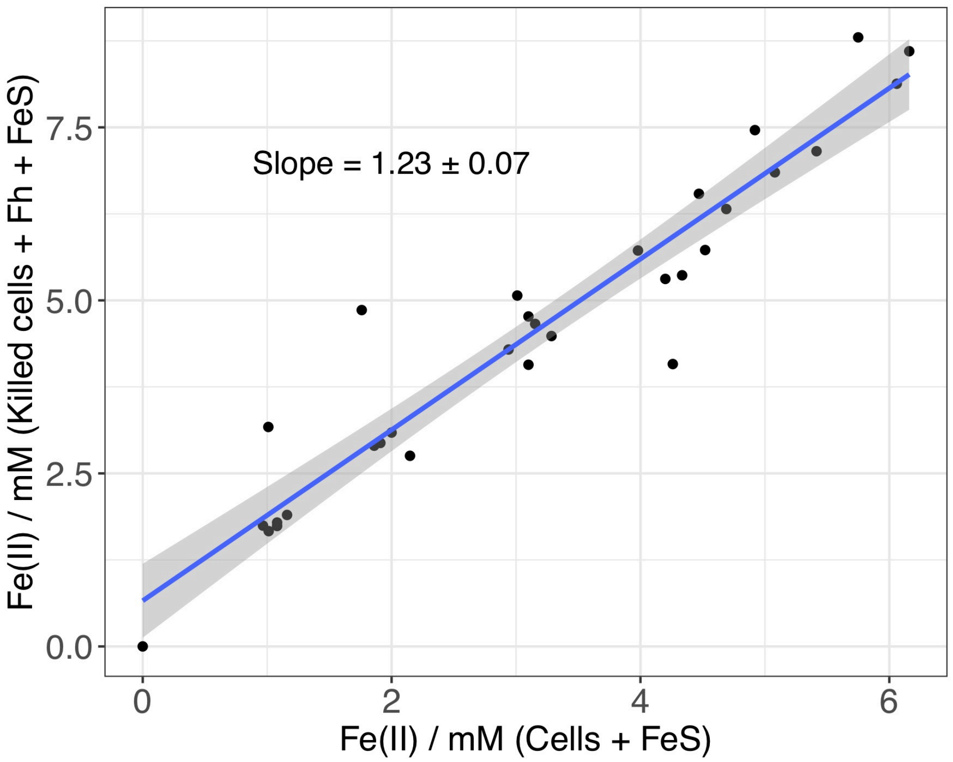

Figure S9 | Overestimation of HCl-extractable Fe(II) in the incubations containing both ferrihydrite and sulfide. The Fe(II) concentrations are from two treatments in the FeS experiment, including (1) the abiotic control fed with both ferrihydrite and FeS and (2) FeS-only control fed with the same amount of FeS. The measured Fe(II) in the FeS-control derived purely from FeS, and in the abiotic control from both FeS and ferrihydrite/sulfide reaction during the acidification step. The slope (mean ± standard deviation) is estimated via linear regression analysis.

Viability of *D. alkaliphilus* in ferrihydrite-only and sulfide-only incubations

To assess the viability of *D. alkaliphilus* in the two control incubations of the experiment with ferrihydrite and sulfide (1 mM), we added formate (10 mM) to the ferrihydrite-only cultures and nitrate (4 mM) to the sulfide-only cultures after 4-day incubation. Continued incubation revealed formate-dependent Fe(II) formation in ferrihydrite-only cultures (Fig. S10a) and nitrate-dependent sulfide consumption in sulfide-only cultures (Fig. S10b). These results confirmed the presence of viable cells in control experiments capable of responding to the addition of an electron donor or acceptor.

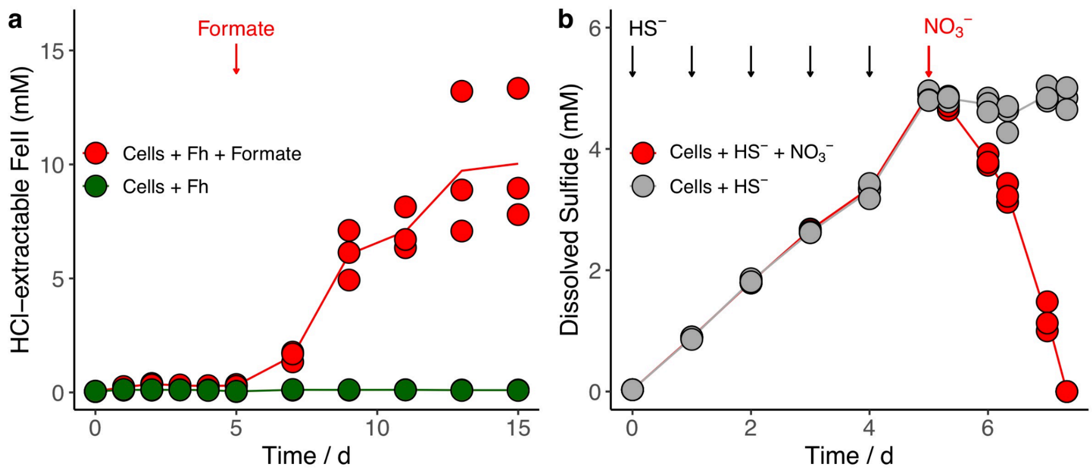

Figure S10 | Viability of *D. alkaliphilus* cells after 4-day incubation with ferrihydrite (ca. 62 mM; Fh) or sulfide alone. a, Formation of HCl-extractable Fe(II) in Fh-only controls upon the addition of 10 mM formate at day 5 confirmed cell viability (red symbols). The arrow indicates the addition of formate. No significant Fe(II) increase was detected in Fh-only controls without formate addition over 15-day

incubation. **b**, Consumption of sulfide in sulfide-only controls upon the addition of 4 mM nitrate at day 5 confirmed cell viability (red symbols). The red arrow indicates the addition of nitrate. Sulfide concentrations did not change in sulfide-only controls without nitrate addition after day 5.

Growth capacity of *D. alkaliphilus*

The growth experiments of *D. alkaliphilus* revealed 2-3 fold increase in cell number over 5-13 days for ferrihydrite-incubated culture amended with either 1 mM sulfide, ca. 50 µM sulfide or FeS (Fig. 4; Fig. S11). In comparison, incubation of *D. alkaliphilus* with sulfide and nitrate led to 5-6 fold increase of cell number over 3 days (Fig. 4; Fig. S11). The generally limited growth capacity seems to reflect an intrinsic property of *D. alkaliphilus* that may be driven by high energy demands for maintenance metabolism. High pH conditions (pH > 9) could increase the maintenance energy requirement, primarily due to the need for cytoplasmic pH homeostasis and the physiological adaptation to environmental stress [31,32]. This elevated maintenance cost likely reduces the fraction of energy available for anabolic reactions, limiting the general growth capacity. The even more constrained growth with ferrihydrite vs. nitrate can be attributed to overall lower energy yield associated with ferrihydrite reduction. Under alkaline conditions tested (pH = 9.3), the ferrihydrite/Fe(II)CO_3 (E^0 = -67.4 mV) has lower standard redox potential compared to the nitrate/ammonium redox couple (E^0 = + 194 mV). This implies ferrihydrite respiration yields less catabolic energy that can be utilized for anabolism, and thereby results in weaker growth compared to nitrate respiration.

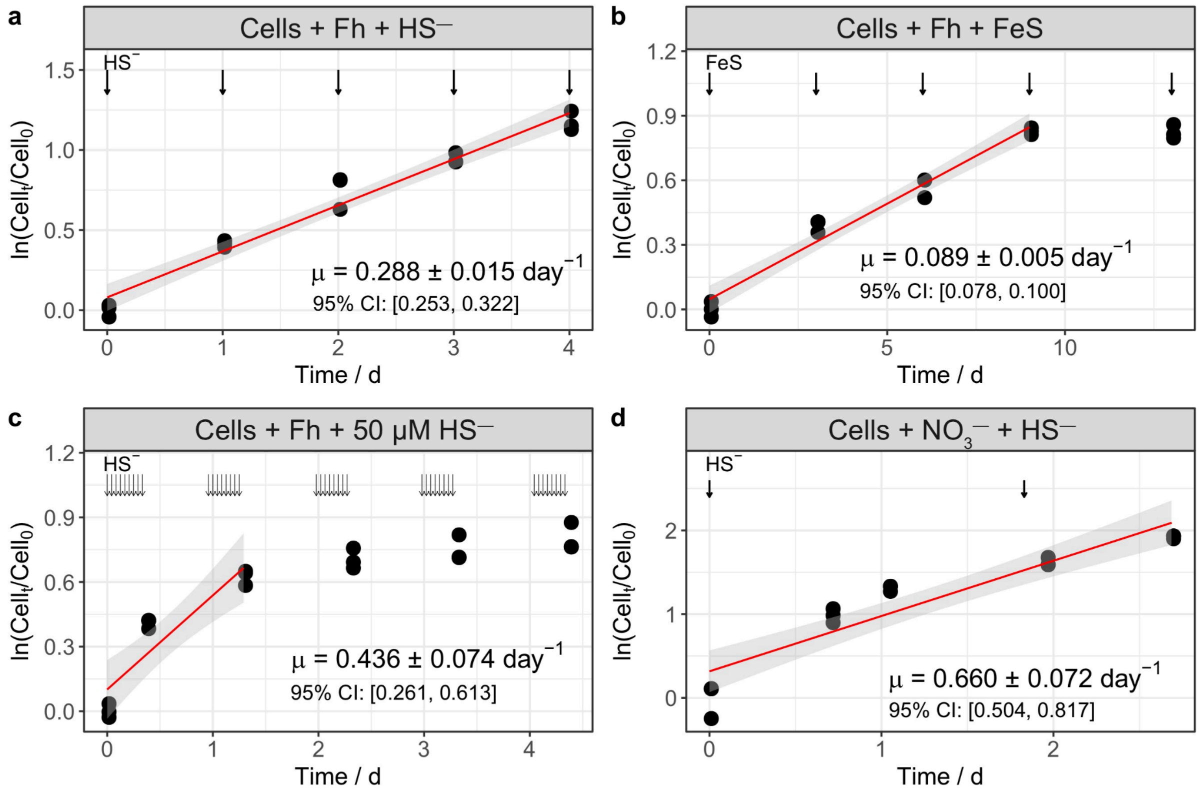

Figure S11 | Specific growth rate (µ; mean ± standard deviation; unit: day^{-1}) of *D. alkaliphilus* with different combinations of electron donors (i.e., sulfide or FeS) and acceptors (ferrihydrite or nitrate). The specific growth rate under each growth condition was estimated by performing linear regression of ln($Cell_t/Cell_0$) on time during the apparent exponential growth phase. $Cell_t$ is the cell concentration (cells ml^{-1}) at sampling time t (in days). The 95% confidence interval (CI) of µ is shown for each growth condition (panel **a**-**d**).

Incubation of *D. alkaliphilus* with ferrihydrite and a small amount of dissolved sulfide

When supplying a small amount of dissolved sulfide (ca. 50 µM) to ferrihydrite-amended *D. alkaliphilus* cultures, we observed rapid consumption of the spiked sulfide (Fig. 3; Extended Data Fig. 1). The estimated sulfide removal rate constant with cells is significantly higher than in abiotic controls (Extended Data Fig. 1). We conclude that the accelerated sulfide consumption is due to biological sulfide oxidation by ferrihydrite, as evidenced by the exclusion of three alternative processes that could support faster sulfide consumption in incubations with cells. First, we ruled out that biological sulfide oxidation by nitrate, potentially carried over from the inoculum, contributed to the observed kinetics. This is supported by negligible sulfide consumption in biotic controls only amended with sulfide (Extended Data Fig. 1b). Second, repeated additions of sulfide to cells resulted in the accumulation of Fe(II) (Extended Data Fig. 1d). Chemical sulfide removal by precipitation with Fe(II) would be expected to result in accelerated sulfide removal rates after consecutive spikes. Yet, the sulfide consumption kinetics after repeated sulfide additions remained similar (Extended Data Fig. 1a and c). This refutes precipitation by Fe(II) as the main sulfide removal process. Third, the chemical reaction between sulfide and ferrihydrite is known to produce surface-associated S(0) [33,34], which may mask the reactive sites of ferrihydrite and limit the chemical reaction rate. The removal of S(0) by its disproportionation by *D. alkaliphilus* could unmask the reactive sites and lead to faster sulfide consumption with cells compared to the abiotic control. We ruled out this possibility considering two aspects: (1) at high ferrihydrite to sulfide molar ratio (>300) as in our incubation, the number of reactive sites on ferrihydrite are not limited and thus surface-coating by S(0) is unlikely restricting the chemical sulfide removal process; and (2) despite accumulation of S(0), sulfide consumption kinetics in chemical controls remained similar over three consecutive additions of sulfide (Extended Data Fig. 1a). This indicates that surface-coating by S(0) is not limiting the chemical reaction rate in our low-sulfide experiments, and the removal of S(0) via disproportionation does not explain the faster sulfide removal in ferrihydrite-amended *D. alkaliphilus* cultures.

We further tracked sulfide turnover in incubations receiving repeated sulfide spikes by measuring concentrations of sulfate, S(0), and Cline-extractable sulfide (Extended Data Fig. 2). Over a series of eight sulfide additions in 1.5-hour intervals, a total of 304.3 ± 4.7 µM dissolved sulfide was supplied to ferrihydrite-amended *D. alkaliphilus* cultures. Within the 12-hour incubation period, the cultures produced 289.3 ± 65.2 µM sulfate and 0.7 ± 0.9 µM S(0), which on average corresponds to 95.1% and 0.2% of the total added sulfide, respectively. These results showed that *D. alkaliphilus* is capable of utilising ferrihydrite to transform the majority of the spiked sulfide to sulfate.

Upregulated transcription of the multi-heme cytochrome gene DA_402 in *D. alkaliphilus* under ferrihydrite-amended conditions

Comparative transcriptomics revealed the transcription of the multi-heme cytochrome gene DA_402 was significantly upregulated in ferrihydrite-amended *D. alkaliphilus* cultures compared to ferrihydrite-free cultures (Fig. S12a). For instance, the transcription level of DA_402 in ferrihydrite-amended cultures with repeated supply of FeS (FeSoxFeR) or dissolved sulfide (SoxFeR) was over 100-folder higher than in cultures that grew by nitrate-dependent sulfide oxidation (SoxNR) or S(0) disproportionation (Sdisp). This transcription pattern was confirmed by quantitative reverse-transcription PCR (Fig. S12b).

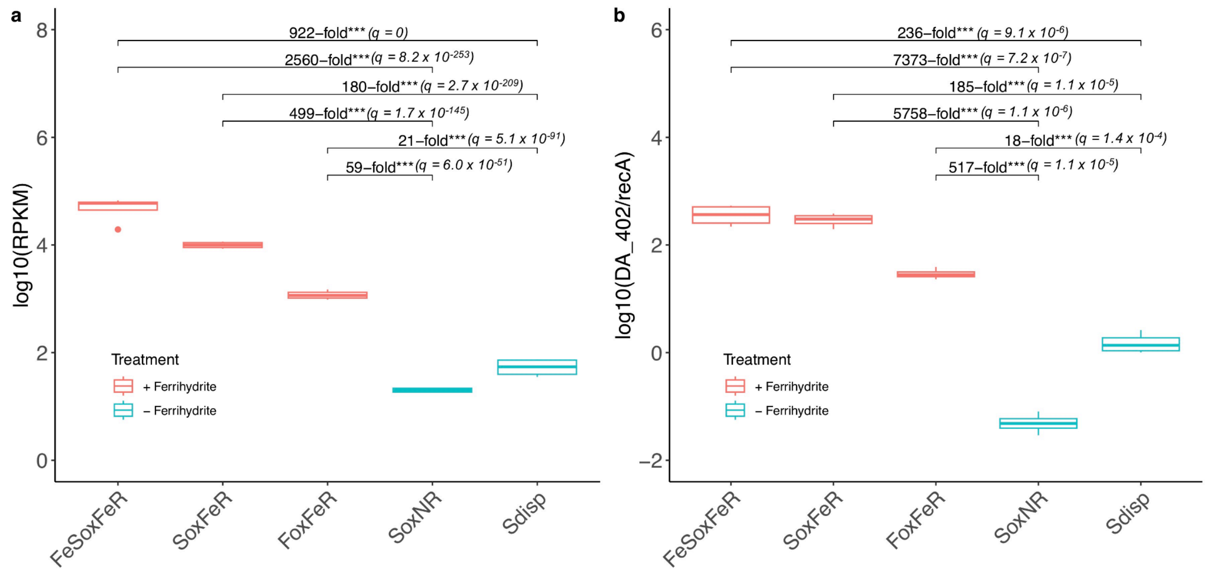

Figure S12 | Transcription level of the multi-heme cytochrome gene DA_402 determined by RNA-Seq (a) or quantitative reverse-transcription PCR (RT-qPCR) (b) under five growth conditions. For RT-qPCR, the transcription level of DA_402 was compared between conditions after normalization with the transcripts of *recA*. For RNA-Seq, differential transcription was assessed by comparing reads per kilobase of transcript per million mapped reads (RPKM). Both RT-qPCR and RNA-Seq showed significantly upregulated transcription of DA_402 in three ferrihydrite-amended conditions (red) compared to two ferrihydrite-free growth conditions (cyan). Up-regulation fold and statistical significance for each of six pairwise comparisons are shown. *** indicates adjusted P (q-values) < 0.001 using the wald-test (a) or the T-test (b) after multiple comparison correction by the Benjamini and Hochberg method. The statistics were derived from four replicate cultures (n = 4) grown under each of five conditions. The centre lines and box limits denote the median, and the 25% and 75% percentile of the transcription levels, respectively, among four replicate cultures of each growth condition. The whiskers extend 1.5 times the interquartile range from the 25th and 75th percentiles. Abbreviation of growth conditions: FeSoxFeR, incubation of *D. alkaliphilus* under FeS-oxidizing and ferrihydrite-reducing conditions. SoxFeR, incubation of *D. alkaliphilus* under sulfide-oxidizing and ferrihydrite-reducing conditions. FoxFeR, incubation of *D. alkaliphilus* under formate-oxidizing and ferrihydrite-reducing conditions. SoxNR, incubation of *D. alkaliphilus* under sulfide-oxidizing and nitrate-reducing conditions. Sdisp, incubation of *D. alkaliphilus* under S(0) disproportionation conditions.

Microscopy of *D. alkaliphilus* incubated with ferrihydrite and sulfide

Scanning electron microscopy (SEM), transmission electron microscopy (TEM), and fluorescence microscopy were performed to image *D. alkaliphilus* incubated with ferrihydrite and sulfide (1 mM). Appendages on the cell outer surfaces were not evident (Fig. 13a-e). We suspect that the absence of nanowire-like structures could be due to (1) the harsh sample preparation procedure, e.g., dithionite treatment to dissolve solid-phase iron, which may have destroyed fragile fine structures like nanowires [35,36], or (2) the possibility that the MHC mediates Fe(III) reduction through direct contact, as demonstrated for other outer membrane cytochromes [37]. The latter is supported by fluorescence microscopy (Fig. 13f), i.e., the observation that most cells were attached to the iron particles and no cells were in the liquid phase.

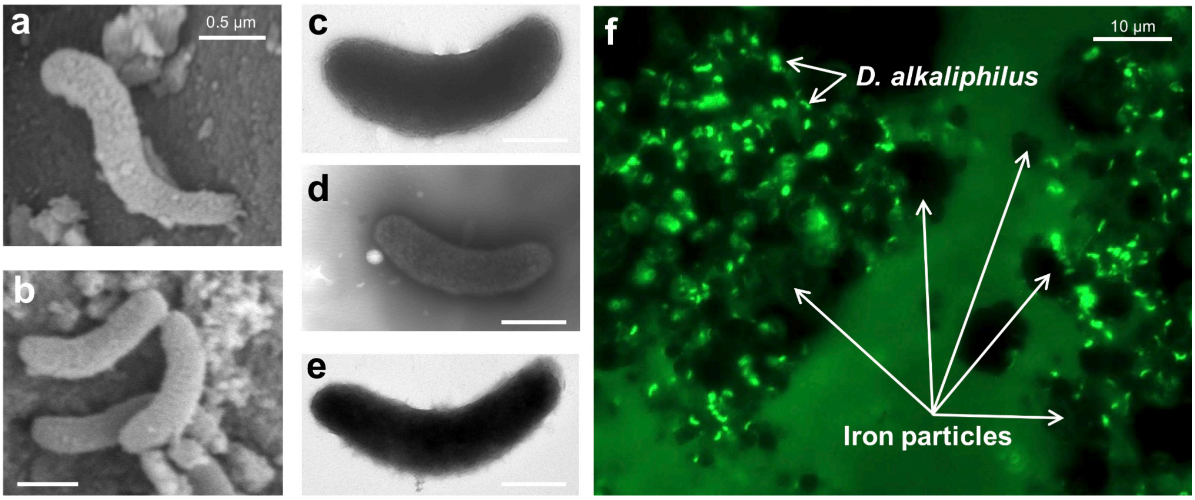

Figure S13 | Microscopic images of *Desulfurivibrio alkaliphilus* grown with ferrihydrite and sulfide. Cells treated with dithionite for removal of solid-phase iron particles were imaged by scanning electron microscopy (**a**, **b**) and transmission electron microscopy (**c**-**e**). The scale bar in panels **a**-**e** shows 0.5 µm. Cells without dithionite treatment were stained with SYBR Green and imaged by fluorescence microscopy (**f**). The arrows point to cells and iron particles. Scanning electron microscopy transmission electron microscopy, and fluorescence microscopy were repeated twice with the same results.

Active transcription of genes involved in Wood Ljungdahl pathway under different growth conditions

Most genes associated with the Wood-Ljungdahl pathway ranked among the top 30% of the most highly transcribed genes in cultures incubated with ferrihydrite and sulfide or FeS (Fig. S14a). A similarly high transcriptional ranking was observed in cultures incubated with either elemental sulfur alone or with formate and ferrihydrite. The highest transcriptional abundance of these genes (ranked top 10%) occurred in the sulfide-oxidizing (Fig. S14b), nitrate-reducing culture, which also showed the greatest growth yield among all tested growth conditions (Fig. 4). These findings suggest the role of the Wood-Ljungdahl pathway in autotrophic carbon fixation.

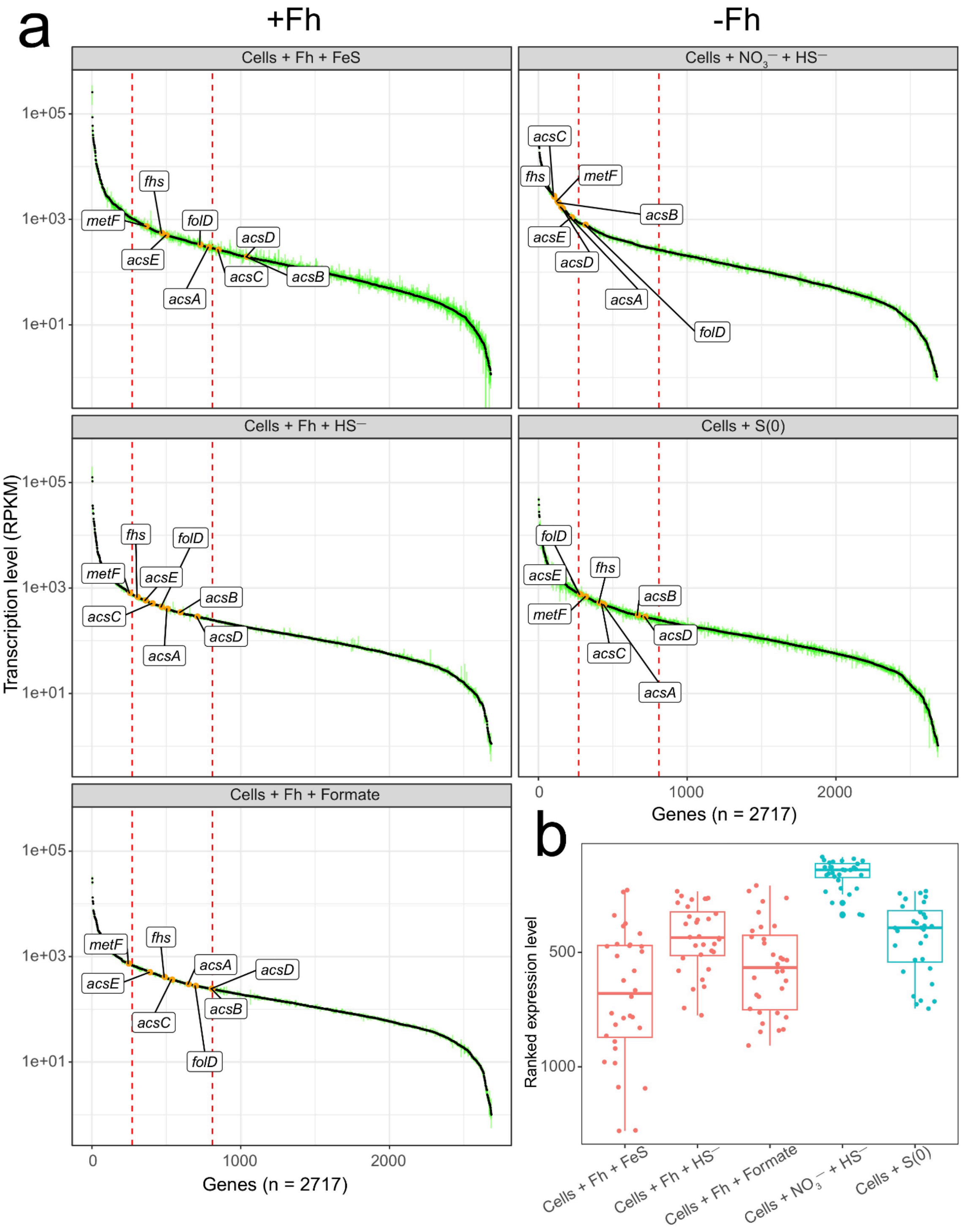

Figure S14 | Ranked transcription abundance plots of *Desulfurivibrio alkaliphilus* genes showing the relative transcription level of Wood Ljungdahl (WL) pathway genes (orange circles) under different growth conditions. a, Each point (black) is the medium transcription level of a gene and error bars (green) correspond to the interquartile range of replicate cultures (n = 4). The two vertical, dashed red lines indicate top 10% and 30% transcribed genes. **b**, Boxplot displaying the ranked transcription abundance of WL genes in three ferrihydrite-amended conditions (red) and two ferrihydrite-free growth conditions (cyan). The center lines and box limits of the boxplot denote the median, and the 25% and 75% percentile of the ranked transcription abundance. The whiskers extend 1.5 times the interquartile range from the 25th and 75th percentiles. Genes involved in WL pathway: *acsA*, acetyl-CoA synthase / CO dehydrogenase (ACS/CODH), complex subunit alpha; *acsB*, ACS/CODH complex subunit beta; *acsC*, ACS/CODH complex subunit gamma; *acsD*, ACS/CODH complex subunit delta; *acsE*, 5-methyltetrahydrofolate corrinoid/iron sulfur protein methyltransferase; *fhs*, formate-tetrahydrofolate ligase;

folD, methenyltetrahydrofolate cyclohydrolase; *metF*, methylenetetrahydrofolate reductase. Fh, ferrihydrite; RPKM, reads per kilobase per million mapped reads.

Gating strategy of flow cytometry

D. alkaliphilus cells were gated based on forward scatter (FSC) and SYBR Green fluorescence using an excitation at 488 nm and emission detection with a 525/40 nm bandpass filter (Fig. S15). Cells were discriminated from background particles by the SYBR Green-induced fluorescence shift relative to unstained (negative) controls. For high throughput analysis a fluorescence threshold was applied on SYBR Green fluorescence to reduce background noise. Total counts were monitored over time, and only data acquired during the period of stable event rates (counts per second) were included in the analysis. Gating was adapted individually for each sample to account for sample-specific variation.

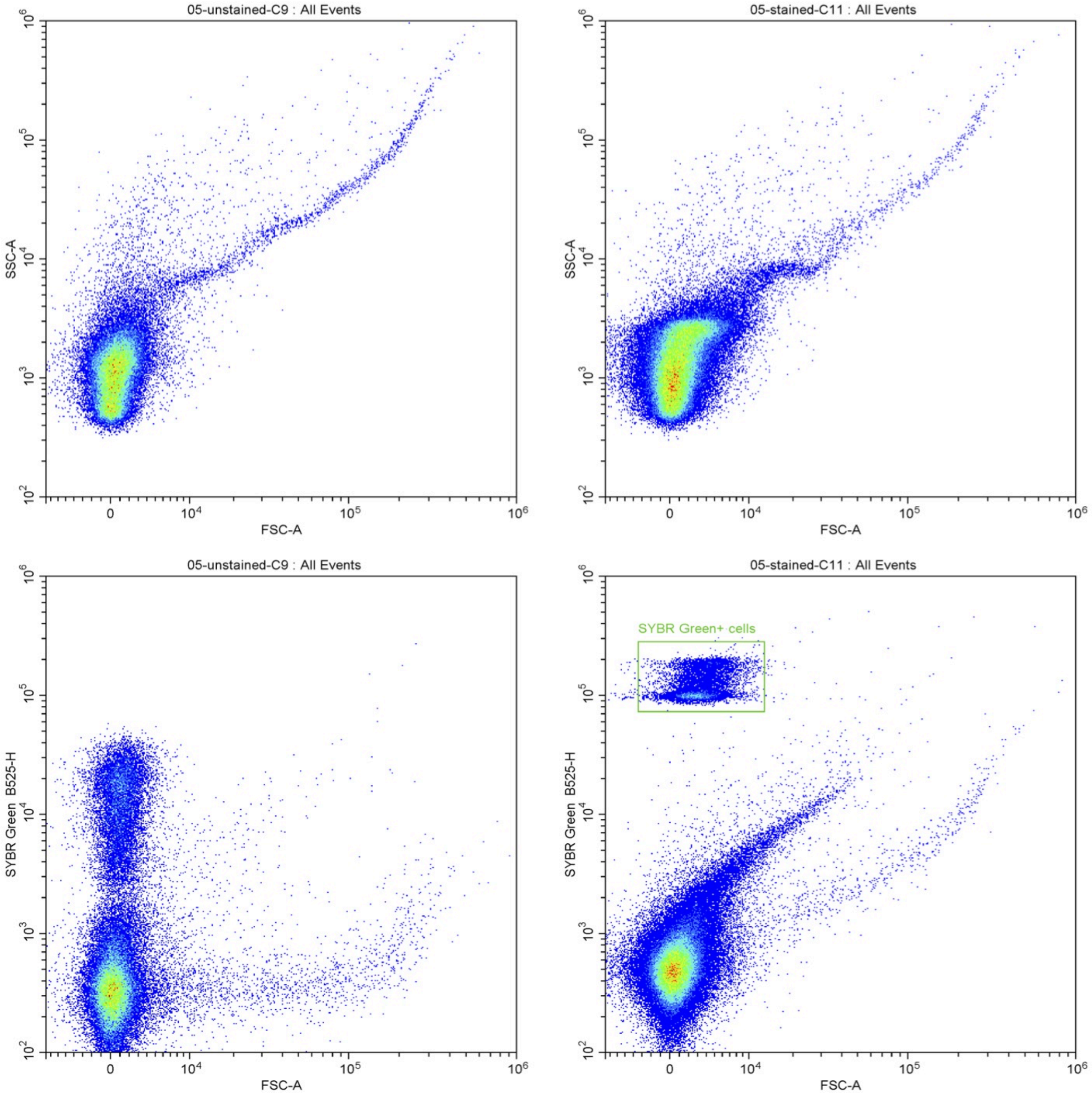

Figure S15 | Gating strategy in flow cytometric cell counting of a *D. alkaliphilus* culture incubated with sulfide and ferrihydrite. Upper panels, Side scatter (SSC) vs. Forward scatter (FSC) of unstained (**a**) and SYBR Green-stained (**b**) subsamples. Lower panels, Green fluorescence vs. FSC in unstained (**c**) and SYBR Green stained (**d**) subsamples using excitation at 488 nm and emission detection with a 525/40 nm bandpass filter. *D. alkaliphilus* cells were identified by their SYBR Green induced fluorescence shift (**d**) relative to unstained controls (**c**). The green gate marks the SYBR Green-stained (SYBR Green+) cells. For subsequent high-throughput measurements a fluorescence threshold was applied on the SYBR Green fluorescence to reduce background noise. Total counts were monitored over time, and only data acquired during periods of stable event rates (counts per second) were included in the analysis. Gate sizes were individually adapted for each sample to account for sample-specific variation.

Cell-specific metabolic rates of MISO

Although overall growth of *D. alkaliphilus* remained modest (Fig. 4; Fig. S11), likely constrained by increased maintenance energy requirements at pH stressed conditions, we observed a high metabolic rate powered by the MISO process. Cell-specific sulfate production rates of MISO reached around 1 fmol SO_4^{2-} $cell^{-1}$ d^{-1} with FeS (Fig. 3b), 4 fmol SO_4^{2-} $cell^{-1}$ d^{-1} with environmentally relevant sulfide (ca. 50 µM; Extended Data Fig. 2), and 16 fmol SO_4^{2-} $cell^{-1}$ d^{-1} with elevated sulfide levels (1 mM; Fig. 3g). For context, these values exceed the cell-specific sulfate reduction rate (csSRR) commonly measured in marine sediments (0.1-5 x 10^{-4} fmol SO_4^{2-} $cell^{-1}$ d^{-1}) by one to four orders of magnitude, and align with csSRR observed in pure cultures of sulfate-reducing bacteria (2-47 fmol SO_4^{2-} $cell^{-1}$ d^{-1}) [38]. Thus, we argue that the MISO process has the potential to influence the biogeochemical sulfur cycle, by producing sulfate at a rate comparable with the sulfate reduction observed in natural environments.

Geochemical phenomena consistent with the activity of MISO

The finding of MISO offers a microbiological explanation for a range of *in situ* phenomena associated with cryptic sulfate formation in anoxic, iron-rich habitats. In marine ecosystems, such phenomena are, for instance, (1) the wide-spread sulfide to sulfate oxidation processes in Arctic coastal and fjord sediments, with the rates being primarily controlled by the quantity and reactivity of iron(III) oxides [39,40], (2) the presence of a physiologically intact community of sulfate-reducing bacteria below the sulfate-methane transition zone in iron-rich marine sediment (Aarhus Bay, Denmark), where sulfate is cryptically formed through strong re-oxidation of sulfide with deeply buried Fe(III) minerals [41,42], and (3) changes of the ^{18}O isotope composition in the sulfate pool indicative of an active sulfate redox cycling, coupled to an enriched ferrous iron concentration and/or strong negative fractionation of ^{56}Fe indicative of dissimilatory iron reduction in salt marsh sediments (Norfolk, England) [43], in sulfate-rich marine brines (beneath Taylor Glacier) [44], and in deep subsurface sediments (Nankai Trough, Japan) [45]. Iron-dependent sulfate formation is also relevant in terrestrial ecosystems. For example, (1) rapid sulfur cycling in low-sulfate (maximally few hundred mM sulfate) wetlands ecosystems is sustained by constant replenishment of the small sulfate pool by re-oxidation of sulfide with poorly crystalline iron oxides as primary oxidants under anoxic conditions [46–51], (2) coincidence of sulfate and ferrous iron peaks along the pore water profile in Fe/Mn accumulated sediment layers in Lake Baikal [45], and (3) transfer of radiolabel from sulfide/FeS to the sulfate pool, concomitant with a build-up of ferrous iron during the incubation of Brabrand Lake sediments with $^{35}HS^-$ [52]. The mechanisms underlying these iron-oxide-associated sulfate formation processes remained unknown. Current geochemical models assert microbial disproportionation is necessary in transforming intermediate sulfur species (e.g., elemental sulfur and thiosulfate) to sulfate [53,54]. Such an interpretation is based on the assumption that the reaction between sulfide and iron(III) oxides is a strictly chemical process that yields predominantly partially oxidized sulfur species. The finding of MISO contrasts this assumption and offers a feasible path to explain cryptic sulfate formation in diverse iron-rich, anoxic environments. This collectively has critical implications for sulfur and iron cycling, carbon mineralization, and methane emission on a global scale [46,54–56].

Relevance of MISO in the global sulfur budget

The oxidation of sulfide, mainly generated by microbial sulfate reduction, is a key process in the global biogeochemical sulfur cycle [39,54,57]. The global estimates of sulfate reduction rate in marine sediments range from 11.3 to 65 Tmol/year, depending on the dataset and models used [58–61]. Mass balance calculations revealed that more than 90% of sulfide, produced from sulfate reduction in marine sediments, is re-oxidized back to sulfate [56,60,62] by using oxidants such as oxygen, nitrate, manganese oxides or iron oxides [54]. This translates to approximately 10.1 to 58.5 Tmol of sulfide being converted to sulfate yearly. To yield a first approximation of the contribution of MISO to global sulfide re-oxidation, we used the global budget of highly reactive iron delivered from rivers to oceans. This iron consists primarily of iron oxides and oxyhydroxide phases (e.g., ferrihydrite and goethite) and is deposited on the seafloor at an estimated rate of 5.5 Tmol/year [63,64]. If the entire flux of reactive iron is used by MISO, which follows an iron:sulfate ratio of 8:1 (Fig. 2a), MISO could

account for 1.1-6.8% of the sulfide-to-sulfate flux in the global seabed. Organic carbon fueled iron reduction and chemical sulfide oxidation may compete with MISO for the reactive iron. However, the potential impact of MISO remains significant given that reactive iron undergoes 100-300 redox cycles before it is permanently buried in the seabed as, e.g., pyrite [65,66].

References

1. Eisen, J. A. Phylogenomics: Improving functional predictions for uncharacterized genes by evolutionary analysis. *Genome Res.* **8**, 163–167 (1998).
2. Aramaki, T. *et al.* KofamKOALA: KEGG Ortholog assignment based on profile HMM and adaptive score threshold. *Bioinformatics* **36**, 2251–2252 (2020).
3. Haft, D. H. *et al.* TIGRFAMs: a protein family resource for the functional identification of proteins. *Nucleic Acids Res.* **29**, 41–43 (2001).
4. Finn, R. D. *et al.* Pfam: The protein families database. *Nucleic Acids Res.* **42**, D222–30 (2014).
5. Neukirchen, S. & Sousa, F. L. DiSCo: A sequence-based type-specific predictor of Dsr-dependent dissimilatory sulphur metabolism in microbial data. *Microb Genom* **7**, (2021).
6. McDaniel, E. A., Anantharaman, K. & McMahon, K. D. metabolisHMM: Phylogenomic analysis for exploration of microbial phylogenies and metabolic pathways. *bioRxiv* (2019) doi:10.1101/2019.12.20.884627.
7. Teng, Z.-J. *et al.* Biogeographic traits of dimethyl sulfide and dimethylsulfoniopropionate cycling in polar oceans. *Microbiome* **9**, 207 (2021).
8. Tanabe, T. S. & Dahl, C. HMS-S-S: A tool for the identification of sulphur metabolism-related genes and analysis of operon structures in genome and metagenome assemblies. *Mol. Ecol. Resour.* **22**, 2758–2774 (2022).
9. Leang, C., Coppi, M. V. & Lovley, D. R. OmcB, a c-type polyheme cytochrome, involved in Fe(III) reduction in *Geobacter sulfurreducens*. *J. Bacteriol.* **185**, 2096–2103 (2003).
10. Liu, Y. *et al.* A trans-outer membrane porin-cytochrome protein complex for extracellular electron transfer by *Geobacter sulfurreducens* PCA: A trans-outer membrane electron transfer complex. *Environ. Microbiol. Rep.* **6**, 776–785 (2014).
11. Liu, Y., Fredrickson, J. K., Zachara, J. M. & Shi, L. Direct involvement of *ombB*, *omaB*, and *omcB* genes in extracellular reduction of Fe(III) by *Geobacter sulfurreducens* PCA. *Front. Microbiol.* **6**, 1075 (2015).
12. Hartshorne, R. S. *et al.* Characterization of an electron conduit between bacteria and the extracellular environment. *Proc. Natl. Acad. Sci. U. S. A.* **106**, 22169–22174 (2009).
13. Light, S. H. *et al.* A flavin-based extracellular electron transfer mechanism in diverse Gram-positive bacteria. *Nature* **562**, 140–144 (2018).
14. Santos, T. C., Silva, M. A., Morgado, L., Dantas, J. M. & Salgueiro, C. A. Diving into the redox properties of *Geobacter sulfurreducens* cytochromes: A model for extracellular electron transfer. *Dalton Trans.* **44**, 9335–9344 (2015).
15. Gupta, D., Chen, K., Elliott, S. J. & Nayak, D. D. MmcA is an electron conduit that facilitates both intracellular and extracellular electron transport in *Methanosarcina acetivorans*. *Nat. Commun.* **15**, 3300 (2024).
16. Deng, X., Dohmae, N., Nealson, K. H., Hashimoto, K. & Okamoto, A. Multi-heme cytochromes provide a pathway for survival in energy-limited environments. *Sci. Adv.* **4**, eaao5682 (2018).
17. Mardanov, A. V. *et al.* The *Geoglobus acetivorans* genome: Fe(III) reduction, acetate utilization, autotrophic growth, and degradation of aromatic compounds in a hyperthermophilic archaeon. *Appl. Environ. Microbiol.* **81**, 1003–1012 (2015).
18. Manzella, M. P., Reguera, G. & Kashefi, K. Extracellular electron transfer to Fe(III) oxides by the hyperthermophilic archaeon *Geoglobus ahangari* via a direct contact mechanism. *Appl. Environ. Microbiol.* **79**, 4694–4700 (2013).
19. Leu, A. O. *et al.* Lateral gene transfer drives metabolic flexibility in the anaerobic methane-oxidizing archaeal family *Methanoperedenaceae*. *mBio* **11**, e02904-21 (2020).
20. Zhang, X. *et al.* Multi-heme cytochrome-mediated extracellular electron transfer by the anaerobic methanotroph *'Candidatus* Methanoperedens nitroreducens'. *Nat. Commun.* **14**, 6118 (2023).
21. Garber, A. I., Nealson, K. H. & Merino, N. Large-scale prediction of outer-membrane multiheme cytochromes uncovers hidden diversity of electroactive bacteria and underlying pathways. *Front. Microbiol.* **15**, 1448685 (2024).
22. Thorup, C., Schramm, A., Findlay, A. J., Finster, K. W. & Schreiber, L. Disguised as a sulfate reducer: Growth of the Deltaproteobacterium *Desulfurivibrio alkaliphilus* by sulfide oxidation with nitrate. *mBio* **8**, e00671-17 (2017).
23. Kjeldsen, K. U. *et al.* On the evolution and physiology of cable bacteria. *Proc. Natl. Acad. Sci. U. S. A.* **116**, 19116–19125 (2019).

24. Shi, L., Fredrickson, J. K. & Zachara, J. M. Genomic analyses of bacterial porin-cytochrome gene clusters. *Front. Microbiol.* **5**, 657 (2014).
25. Portela, P. C. *et al.* Widespread extracellular electron transfer pathways for charging microbial cytochrome OmcS nanowires via periplasmic cytochromes PpcABCDE. *Nat. Commun.* **15**, 2434 (2024).
26. Finneran, K. T., Johnsen, C. V. & Lovley, D. R. *Rhodoferax ferrireducens* sp. nov., a psychrotolerant, facultatively anaerobic bacterium that oxidizes acetate with the reduction of Fe(III). *Int. J. Syst. Evol. Microbiol.* **53**, 669–673 (2003).
27. Baker, I. R., Conley, B. E., Gralnick, J. A. & Girguis, P. R. Evidence for horizontal and vertical transmission of Mtr-mediated extracellular electron transfer among the bacteria. *mBio* **13**, e02904-21 (2021).
28. Marcia, M., Ermler, U., Peng, G. & Michel, H. A new structure-based classification of sulfide:quinone oxidoreductases. *Proteins* **78**, 1073–1083 (2010).
29. Gregersen, L. H., Bryant, D. A. & Frigaard, N.-U. Mechanisms and evolution of oxidative sulfur metabolism in green sulfur bacteria. *Front. Microbiol.* **2**, 116 (2011).
30. Peiffer, S. *et al.* Pyrite formation and mineral transformation pathways upon sulfidation of ferric hydroxides depend on mineral type and sulfide concentration. *Chem. Geol.* **400**, 44–55 (2015).
31. Krulwich, T. A. Alkaliphiles: 'basic' molecular problems of pH tolerance and bioenergetics. *Mol. Microbiol.* **15**, 403–410 (1995).
32. Padan, E., Bibi, E., Ito, M. & Krulwich, T. A. Alkaline pH homeostasis in bacteria: New insights. *Biochim. Biophys. Acta* **1717**, 67–88 (2005).
33. Kumar, N., Lezama Pacheco, J., Noël, V., Dublet, G. & Brown, G. E. Sulfidation mechanisms of Fe(III)-(oxyhydr)oxide nanoparticles: a spectroscopic study. *Environ. Sci. Nano* **5**, 1012–1026 (2018).
34. He, L. *et al.* Elucidating the role of sulfide on the stability of ferrihydrite colloids under anoxic conditions. *Environ. Sci. Technol.* **53**, 4173–4184 (2019).
35. Ray, R., Lizewski, S., Fitzgerald, L. A., Little, B. & Ringeisen, B. R. Methods for imaging *Shewanella oneidensis* MR-1 nanofilaments. *J. Microbiol. Methods* **82**, 187–191 (2010).
36. Gorby, Y. A. *et al.* Electrically conductive bacterial nanowires produced by *Shewanella oneidensis* strain MR-1 and other microorganisms. *Proc. Natl. Acad. Sci. U. S. A.* **103**, 11358–11363 (2006).
37. Shi, L. *et al.* The roles of outer membrane cytochromes of *Shewanella* and *Geobacter* in extracellular electron transfer. *Environ. Microbiol. Rep.* **1**, (2009).
38. Hoehler, T. M. & Jørgensen, B. B. Microbial life under extreme energy limitation. *Nat. Rev. Microbiol.* **11**, 83–94 (2013).
39. Findlay, A. J., Pellerin, A., Laufer, K. & Jørgensen, B. B. Quantification of sulphide oxidation rates in marine sediment. *Geochim. Cosmochim. Acta* **280**, 441–452 (2020).
40. Michaud, A. B. *et al.* Glacial influence on the iron and sulfur cycles in Arctic fjord sediments (Svalbard). *Geochim. Cosmochim. Acta* **280**, 423–440 (2020).
41. Pellerin, A. *et al.* The sulfur cycle below the sulfate-methane transition of marine sediments. *Geochim. Cosmochim. Acta* **239**, 74–89 (2018).
42. Holmkvist, L., Ferdelman, T. G. & Jørgensen, B. B. A cryptic sulfur cycle driven by iron in the methane zone of marine sediment (Aarhus Bay, Denmark). *Geochim. Cosmochim. Acta* **75**, 3581–3599 (2011).
43. Mills, J. V., Antler, G. & Turchyn, A. V. Geochemical evidence for cryptic sulfur cycling in salt marsh sediments. *Earth Planet. Sci. Lett.* **453**, 23–32 (2016).
44. Mikucki, J. A. *et al.* A contemporary microbially maintained subglacial ferrous 'ocean'. *Science* **324**, 397–400 (2009).
45. Och, L. M. *et al.* New insights into the formation and burial of Fe/Mn accumulations in Lake Baikal sediments. *Chem. Geol.* **330-331**, 244–259 (2012).
46. Pester, M., Knorr, K.-H., Friedrich, M. W., Wagner, M. & Loy, A. Sulfate-reducing microorganisms in wetlands - fameless actors in carbon cycling and climate change. *Front. Microbiol.* **3**, 72 (2012).
47. Knorr, K.-H. & Blodau, C. Impact of experimental drought and rewetting on redox transformations and methanogenesis in mesocosms of a northern fen soil. *Soil Biol. Biochem.* **41**, 1187–1198 (2009).
48. Knorr, K.-H., Lischeid, G. & Blodau, C. Dynamics of redox processes in a minerotrophic fen exposed to a water table manipulation. *Geoderma* **153**, 379–392 (2009).
49. Pester, M., Bittner, N., Deevong, P., Wagner, M. & Loy, A. A 'rare biosphere' microorganism contributes to sulfate reduction in a peatland. *ISME J.* **4**, 1591–1602 (2010).
50. Reiche, M., Torburg, G. & Küsel, K. Competition of Fe(III) reduction and methanogenesis in an acidic fen. *FEMS Microbiol. Ecol.* **65**, 88–101 (2008).
51. Guo, W., Cecchetti, A. R., Wen, Y., Zhou, Q. & Sedlak, D. L. Sulfur cycle in a wetland microcosm: Extended ^{34}S-stable isotope analysis and mass balance. *Environ. Sci. Technol.* **54**, 5498–5508 (2020).

52. Elsgaard, L. & Jørgensen, B. B. Anoxic transformations of radiolabeled hydrogen sulfide in marine and freshwater sediments. *Geochim. Cosmochim. Acta* **56**, 2425–2435 (1992).
53. Thamdrup, B., Finster, K., Hansen, J. W. & Bak, F. Bacterial disproportionation of elemental sulfur coupled to chemical reduction of iron or manganese. *Appl. Environ. Microbiol.* **59**, 101–108 (1993).
54. Jørgensen, B. B., Findlay, A. J. & Pellerin, A. The biogeochemical sulfur cycle of marine sediments. *Front. Microbiol.* **10**, 849 (2019).
55. Jørgensen, B. B. Mineralization of organic matter in the sea bed—the role of sulphate reduction. *Nature* **296**, 643–645 (1982).
56. Jørgensen, B. B., Bang, M. & Blackburn, T. H. Anaerobic mineralization in marine sediments from the Baltic Sea-North Sea transition. *Mar. Ecol. Prog. Ser.* **59**, 39–54 (1990).
57. Jørgensen, B. B. A thiosulfate shunt in the sulfur cycle of marine sediments. *Science* **249**, 152–154 (1990).
58. Canfield, D. E., Kristensen, E. & Thamdrup, B. Aquatic geomicrobiology. *Adv. Mar. Biol.* **48**, 1–599 (2005).
59. Bowles, M. W., Mogollón, J. M., Kasten, S., Zabel, M. & Hinrichs, K.-U. Global rates of marine sulfate reduction and implications for sub-sea-floor metabolic activities. *Science* **344**, 889–891 (2014).
60. Barker Jørgensen, B., Egger, M. & Canfield, D. E. Sulfate distribution and sulfate reduction in global marine sediments. *Geochim. Cosmochim. Acta* **364**, 79–88 (2024).
61. Jørgensen, B. B. & Kasten, S. Sulfur cycling and methane oxidation. in *Marine Geochemistry* 271–309 (Springer-Verlag, Berlin/Heidelberg, 2006).
62. Thamdrup, B., Fossing, H. & Jørgensen, B. B. Manganese, iron and sulfur cycling in a coastal marine sediment, Aarhus bay, Denmark. *Geochim. Cosmochim. Acta* **58**, 5115–5129 (1994).
63. Poulton, S. W. The low-temperature geochemical cycle of iron: From continental fluxes to marine sediment deposition. *Am. J. Sci.* **302**, 774–805 (2002).
64. Raiswell, R. & Canfield, D. E. The iron biogeochemical cycle past and present. *Geochem. Perspect.* **1**, 1–220 (2012).
65. Canfield, D. E., Thamdrup, B. & Hansen, J. W. The anaerobic degradation of organic matter in Danish coastal sediments: Iron reduction, manganese reduction, and sulfate reduction. *Geochim. Cosmochim. Acta* **57**, 3867–3883 (1993).
66. Beal, E. J., House, C. H. & Orphan, V. J. Manganese- and iron-dependent marine methane oxidation. *Science* **325**, 184–187 (2009).
